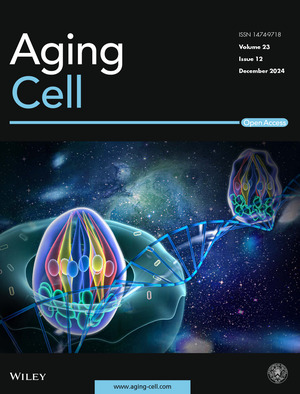# Additional Cover

**DOI:** 10.1111/acel.14455

**Published:** 2024-12-11

**Authors:** Wenwen Ren, Weihao Li, Xudong Cha, Shenglei Wang, Boyu Cai, Tianyu Wang, Fengzhen Li, Tengfei Li, Yingqi Xie, Zengyi Xu, Zhe Wang, Huanhai Liu, Yiqun Yu

## Abstract

Cover legend: The cover image is based on the article *Single‐cell transcriptomic atlas of taste papilla aging* by Yiqun Yu et al., https://doi.org/10.1111/acel.14308.